# From metabolism to mood regulation: astrocytes as a driver of depression

**DOI:** 10.3389/fncel.2026.1776649

**Published:** 2026-03-04

**Authors:** Yusuke Nasu, Miho Terunuma

**Affiliations:** 1 Division of Oral Biochemistry, Faculty of Dentistry, Graduate School of Medical and Dental Sciences, Niigata University, Niigata, Japan; 2 Division of Periodontology, Faculty of Dentistry, Graduate School of Medical and Dental Sciences, Niigata University, Niigata, Japan

**Keywords:** animal model, astrocytes, depression, gain-of-function, loss-of-function

## Abstract

Astrocytes are increasingly recognized as active regulators of mood and cognition, extending far beyond their classical supportive roles. In major depressive disorder, converging evidence from postmortem analyses, magnetic resonance spectroscopy (MRS), and animal stress models points toward the possibility of astrocytic abnormalities, including reduced density, impaired glutamate–glutamine cycling, and altered mitochondrial function. However, the causal contribution of these alterations remains insufficiently defined. This review aims to summarize experimental studies employing both loss- and gain-of-function approaches to directly probe the involvement of astrocytes in depression. We first introduce which inhibited astrocytic functions induce depressive-like behaviors, and then explore how enhancing these astrocytic functions—through overexpression and pharmacological manipulation methods—rescues stress-induced depression phenotypes. We further connect astrocyte alterations with circuit-level dysfunctions and behavioral outcomes, such as impaired prefrontal–amygdala regulation and reduced mesolimbic reward responses. Finally, we discuss therapeutic opportunities including astrocyte-targeting pharmacological strategies and MRS-based biomarkers. By integrating mechanistic evidence with translational perspectives, this review positions astrocyte metabolism as a promising frontier for antidepressant development.

## 1 Introduction

Major depressive disorder (MDD) could affect more than 300 million people worldwide (World Health Organization, 2017) and imposes substantial socioeconomic burdens through suicide, reduced productivity, and impaired work capacity (Arias-de la Torre et al., 2021; James et al., 2018; Santomauro et al., 2021). Although the monoamine hypothesis, which centers on monoaminergic neurotransmitter depletion and receptor dysfunction, has historically dominated as biological models of MDD pathophysiology (Hillhouse and Porter, 2015), the delayed therapeutic onset of antidepressants and the prevalence of treatment-resistant cases highlight the limitations of this biomedical framework (Berlim and Turecki, 2007; Pastis et al., 2024).

**Figure 1:**
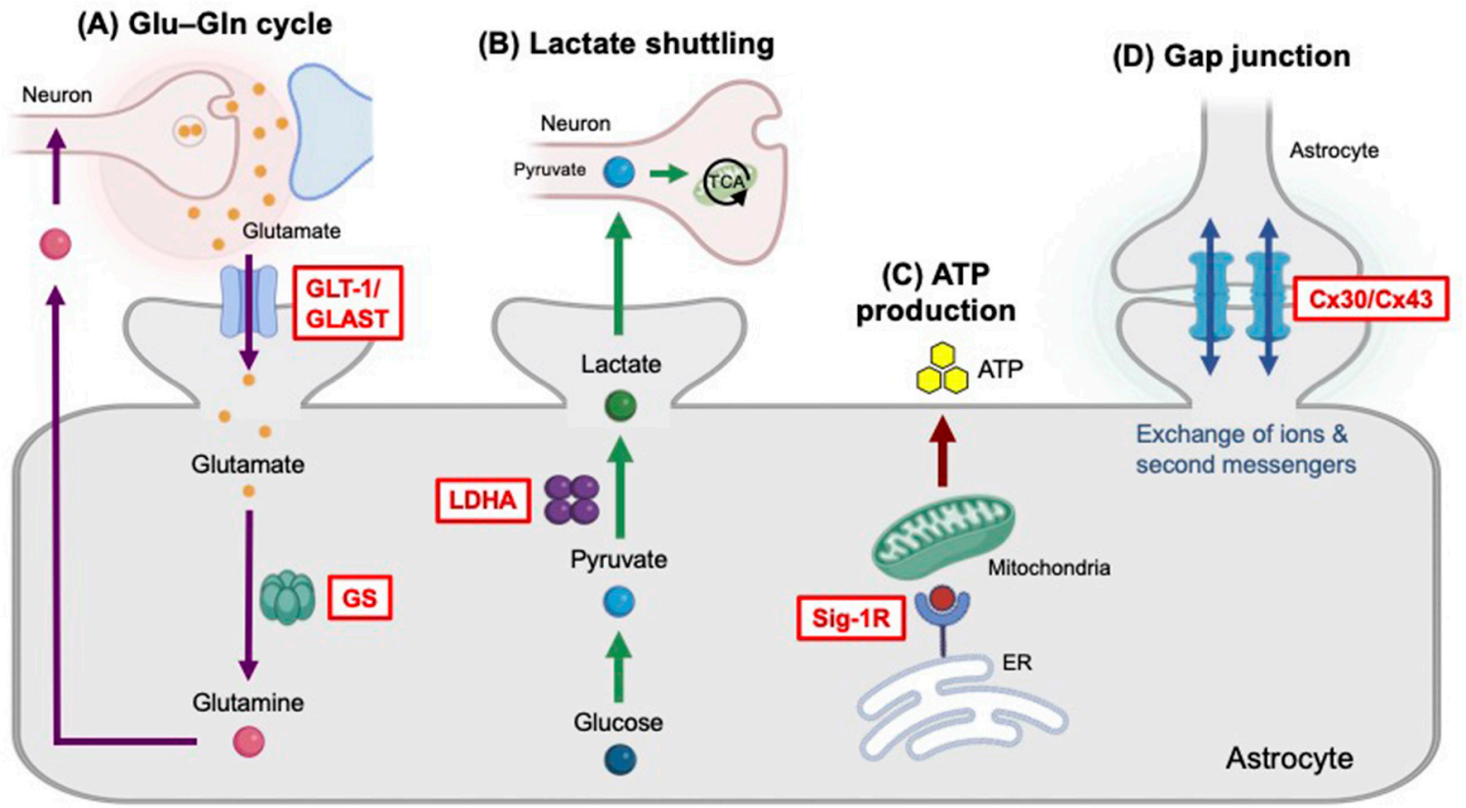
Selected astrocytic functions important for mood regulation. **(A)** The Glu–Gln cycle is a vital astrocyte–neuron metabolic pathway that prevents excitotoxicity by recycling neurotransmitters. The Glu transporters GLT-1 and GLAST are responsible for clearing Glu from the synaptic cleft. Once inside the astrocyte, the enzyme GS converts Glu and ammonia into Gln, a process essential for both neurotransmitter recycling and ammonia detoxification. Finally, this Gln is transported out of the astrocyte, via SNAT3/5, and is subsequently taken up by neurons to be converted back into Glu. **(B)** The astrocyte–neuron lactate shuttling is a well-established model of metabolic relationship in the brain, where astrocytes metabolize glucose to lactate—utilizing the enzyme LDHA—and export it to neurons to fuel their high energy demands. **(C)** Astrocytic Sig-1R critically regulates mitochondrial function under stress by maintaining ER-mitochondrial homeostasis and Ca^2+^ signaling, thereby modulating mitochondrial respiration and ATP production. ATP released from astrocytes acts to influence neuronal circuits. **(D)** Astrocyte gap junctions connect neighboring astrocytes, allowing them to form a vast interconnected functional network. These junctions facilitate the passage of ions, small metabolites, and second messengers.

Increasing evidence now points to astrocytes, star-shaped glial cells that play key roles in the regulation of synaptic transmission and plasticity, gliotransmission, brain energy metabolism, and neural circuit homeostasis, as essential contributor of emotional regulation (Cui et al., 2024; Lyon and Allen, 2022). Postmortem studies in patients with MDD have consistently reported structural changes in the brain, featuring reduced densities and morphological atrophy of glial fibrillary acidic protein (GFAP)-positive astrocytes in the prefrontal cortex (PFC) and hippocampus (Czéh and Nagy, 2018; Miguel-Hidalgo et al., 2000; Qi et al., 2019; Si et al., 2004), along with lower levels of reactive astrogliosis in regions such as the thalamus and caudate nucleus (Torres-Platas et al., 2016). Magnetic resonance spectroscopy (MRS) studies in MDD patients have revealed reductions in the glutamate (Glu)-to-glutamine (Gln) ratio, combined Glx (Glu + Gln) levels, and myo-inositol levels (Block et al., 2009; Coupland et al., 2005; Moriguchi et al., 2019). These findings suggest dysfunction of the astrocytic Glu–Gln cycle, astrocyte metabolism, and osmotic regulation, although results often vary by brain region (Luykx et al., 2012). Supporting these observations, the studies in animal models of chronic stress have demonstrated astrocyte abnormalities including reductions in GFAP expression, impaired Glu–Gln metabolism and lactate shuttling, mitochondrial dysfunction, and compromised gap junction coupling (Gosselin et al., 2009; Murphy-Royal et al., 2020; Shu et al., 2019; Sun et al., 2012; Yao et al., 2023; Figure 1). However, a central unresolved question remains whether astrocytic alterations represent the cause or the consequence of the pathophysiology of depression.

In this review, we summarize the existing literature that directly manipulates astrocyte function in rodents using loss-of-function (LOF) and gain-of-function (GOF) methods to understand the involvement of astrocytes in the pathophysiological mechanism of depression. Furthermore, we describe how astrocytic abnormalities affect neural circuits—such as PFC-amygdala dysregulation and diminished mesolimbic reward responses—and discuss the therapeutic opportunities of targeting astrocytes.

## 2 Loss-of-function studies in astrocytes

**Figure 2:**
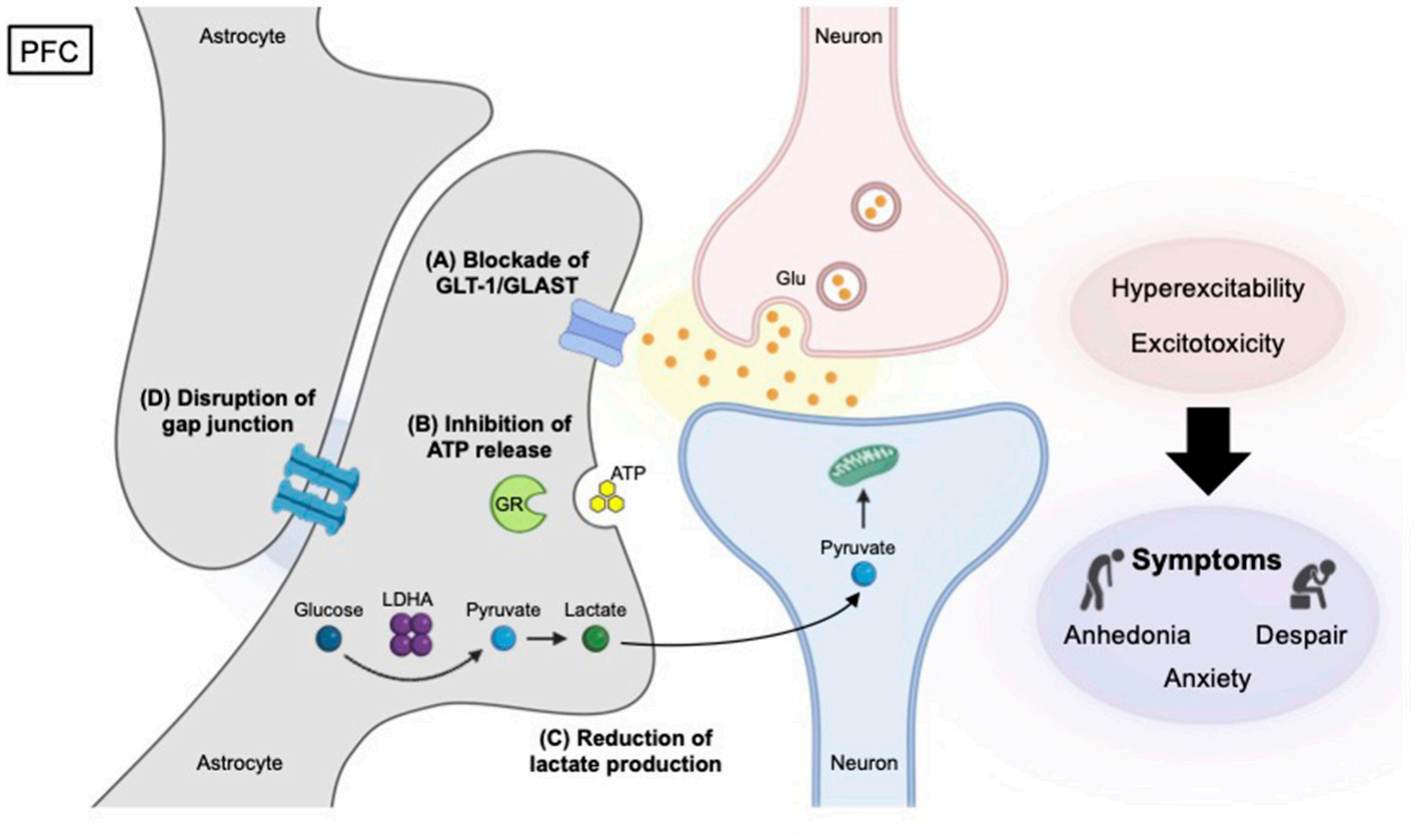
Represented causal mechanisms of depression identified by LOF studies in the PFC. Reduced GLT-1/GLAST function **(A)** disrupts Glu-Gln cycle, leading to excessive synaptic Glu accumulation, excitotoxicity, impaired synaptic plasticity, and depression. Diminished ATP release **(B)** from astrocytes caused by the deletion of the glucocorticoid receptor (GR) gene (Nr3cl) induces depression primarily by disrupting essential astrocyte-neuron communication and increasing neuronal activity. Disturbance of Cx30/Cx43 gap junctions **(C)** in astrocytes dismantles the functional syncytium—a large-scale interconnected network of astrocytes—leading to ionic imbalances, neuronal hyperexcitability, and depressive-like behaviors. In contrast, a reduction in lactate production **(D)** through the elimination of glycolytic enzyme LDHA causes depression by reducing neuronal excitability, suggesting that astrocyte-derived lactate is critical for supporting normal neuronal activity.

LOF approaches, which involve reducing or eliminating the activity of specific genes or proteins, have significantly advanced the understanding of the underlying biology of depression. These approaches often provide causal evidence that impairments in astrocytic functions disrupt neuronal activity and drive depression-related phenotypes, thereby establishing these pathways as critical substrates for stress vulnerability and mood dysregulation (Figure 2 and Table 1). This chapter introduces the importance of Glu clearance, adenosine triphosphate (ATP) signaling, lactate production, calcium entry, potassium buffering, and gap junctions in astrocytes.

**Table 1:** LOF/GOF studies targeting astrocytes to understand the molecular underpinnings of depression.

**Target molecules**	**LOF or GOF**	**Manipulated brain area**	**Phenotype**	**Species**	**References**
ALKBH5	LOF	mPFC	Anhedonia ↓ Despair ↓ Social withdrawal ↓	Mouse	Guo et al., 2024
	GOF	mPFC	Despair ↑ Social withdrawal ↑	Mouse	Guo et al., 2024
AQP4	LOF	Hippocampus	Despair ↑	Mouse	Kong et al., 2014
Cx30, Cx43	LOF	mPFC, hippocampus	Despair ↑	Mouse	Huang et al., 2019
	GOF	mPFC, hippocampus	Anxiety ↓ Despair ↓	Mouse	Huang et al., 2019
Cx43	LOF	PFC	Anhedonia ↑ Anxiety ↑ Despair ↑	Mouse	Sun et al., 2012
Soluble epoxide hydrolase	LOF	mPFC	Anhedonia ↓ Despair ↓ Social withdrawal ↓	Mouse	Xiong et al., 2019
FABP7	GOF	Hippocampus	Anhedonia ↓ Despair ↓	Mouse	Geng et al., 2025
GLT-1	LOF	CeA	Anxiety ↑ Anhedonia ↑	Rat	John et al., 2015
		PFC	Anhedonia ↑	Rat	John et al., 2012
		PFC	Anhedonia ↑	Rat	John et al., 2015
		Whole brain, mPFC	Anhedonia ↑	Rat	Bechtholt-Gompf et al., 2010
	GOF	PFC, hippocampus	Anhedonia ↓ Despair ↓	Rat	Banasr et al., 2010
GLT-1/GLAST	LOF	mPFC	Anhedonia ↑ Anxiety ↑ Despair ↑	Mouse	Fullana et al., 2019
	GOF	vHIP	Anhedonia ↓ Despair ↓	Mouse	Tsai et al., 2022
Glucocorticoid receptor	LOF	mPFC	Anxiety ↑ Despair ↑ Social withdrawal ↑	Mouse	Lu et al., 2022
GS	LOF	PLC	Despair ↑	Mouse	Lee et al., 2013
	GOF	mPFC	Anhedonia ↓ Despair ↓	Mouse	Kang et al., 2025
Insulin receptor	LOF	Whole brain, NAc	Anhedonia ↑ Anxiety ↑ Despair ↑	Mouse	Cai et al., 2018
IP3R2	LOF	NAc	Anhedonia ↑ Despair ↑ Grooming ↓	Mouse	Cao et al., 2013
Kir6.1/K-ATP channel	LOF	Hippocampus	Anhedonia ↑ Despair ↑ Social withdrawal ↑	Mouse	Li et al., 2022
Kir4.1	LOF	LHb	Anhedonia ↓ Despair ↓	Rat	Cui et al., 2018
LDHA	LOF	dmPFC	Anhedonia ↑ Despair ↑ Social withdrawal ↑	Mouse	Yao et al., 2023
O-GlcNAc transferase	LOF	mPFC	Anhedonia ↓ Despair ↓ Social withdrawal ↓	Mouse	Fan et al., 2023
Orai1	LOF	Hippocampus (CA1)	Anhedonia ↓ Despair ↓	Mouse	Novakovic et al., 2023
P2X7 receptor	LOF	Hippocampus (CA1)	Despair ↑	Mouse	Cheng et al., 2025
PTN	LOF	mPFC	Anhedonia ↑ Anxiety ↑ Despair ↑	Mouse	Chi et al., 2025
	GOF	dmPFC	Anhedonia ↓ Anxiety ↓ Despair ↓	Mouse	Chi et al., 2025
Sig-1R	LOF	vHIP	Anhedonia ↑ Anxiety ↑ Despair ↑	Mouse	Li et al., 2025
	GOF	Hippocampus, cortex	Anhedonia ↓ Despair ↓	Mouse	Guo et al., 2021
		vHIP	Anhedonia ↓ Anxiety ↓ Despair ↓	Mouse	Li et al., 2025
TAMM41	LOF	Hippocampus	Anhedonia ↑ Anxiety ↑ Despair ↑	Mouse	Guo et al., 2025
VNUT	LOF	NAc	Anhedonia ↑ Anxiety ↑	Mouse	Huang et al., 2025
	GOF	Hippocampus	Despair ↓	Mouse	Kinoshita et al., 2018

CeA, central nucleus of the amygdala; dmPFC, dorsomedial prefrontal cortex; mPFC, medial prefrontal cortex; NAc, nucleus accumbens; vHIP, ventral hippocampus; ↑ indicates an increase and ↓ indicates a decrease of behavioral phenotype compared with controls.

### 2.1 Blockade of glutamate clearance in astrocytes

Astrocytes respond to extracellular Glu released from neurons and glial cells, and astrocyte-mediated neuroprotection against excitotoxicity is conferred by the clearance of Glu from the extracellular space. Selective astrocytic damage induced by the gliotoxin L-α-aminoadipic acid (L-AAA) in the PFC elicits depression-like behavior (Banasr and Duman, 2008) and significantly lowers Glu and Gln levels in the PFC (Lee et al., 2013). In fact, L-AAA-treated astrocytes induce neuronal atrophy and synapse loss, which can be prevented by the inhibition of the N-methyl-D-aspartate (NMDA)-nitric oxide signaling pathway (David et al., 2018).

Astrocytes are indispensable for maintaining both excitatory neurotransmission and the Glu–Gln cycle. Inhibition of the major astrocytic Glu transporter GLT-1 (EAAT2) induces depression-like behavior. Local administration of GLT-1 inhibitor dihydrokainic acid (DHK) into the lateral ventricle or the medial prefrontal cortex (mPFC) reduces reward sensitivity and induces anhedonia-like behavior, suggesting the induction of depressive-like states (Bechtholt-Gompf et al., 2010). Furthermore, knockdown of another astrocytic Glu transporter GLAST (EAAT1) and GLT-1 in the infralimbic (IL) cortex—achieved by unilateral microinfusion of small interfering RNA (siRNA)—produces depression-like phenotypes characterized by increased neuronal activity in the IL and reduced brain-derived neurotrophic factor (BDNF) mRNA levels (Fullana et al., 2019). Diminished BDNF levels are strongly corelated with depression, as they interact with serotonin and other neurotransmitter systems crucial for mood regulation (Martinowich and Lu, 2008).

Water channel function is also involved in maintaining Glu clearance and homeostasis. In a repeated corticosterone injection model of depression, gene knockout of the astrocytic water channel aquaporin-4 (AQP4^−/−^ mice), found to reduce GLAST and GLT-1 expression, disrupt Glu clearance, and exacerbate depression-like behavior (Kong et al., 2014). Furthermore, AQP4 elimination causes a reduction in GFAP^+^ cell density, and aggravates impairments in corticosterone-induced hippocampal neurogenesis, with a marked reduction in doublecortin-positive immature neuron density (Kong et al., 2014).

### 2.2 Inhibition of astrocyte-mediated ATP signaling

Astrocytes contain a millimolar concentration of ATP, which is constantly generated from adenosine diphosphate (ADP) and adenosine monophosphate (AMP) by the cellular respiration machinery. Astrocytic ATP is a fundamental energy currency for tasks such as ion buffering and neurotransmitter recycling. ATP released from astrocytes, along with its breakdown product adenosine, modulates neuronal function by regulating synaptic plasticity, neurotransmitter release and neuronal metabolism. Astrocytes express purinergic receptors (P2X and P2Y types), creating an autocrine loop to self-regulate various cellular responses involved in brain homeostasis. Additionally, ATP-sensitive potassium (K-ATP) channels in astrocytes act as metabolic sensors, linking cellular energy status (the ATP/ADP ratio) to membrane potential.

#### 2.2.1 Molecules regulating ATP exocytosis from astrocytes

ATP released from astrocytes is a key gliotransmitter that modulates depression-like behavior. The astrocyte-specific removal of the vesicular nucleotide transporter VNUT—achieved by crossing VNUT^flox/flox^ mice with GFAP-CreER^T2^ or Aldh1l1-CreER^T2^ mice and administering tamoxifen—diminishes ATP secretion by approximately 50%, resulting in reduced motivational drive and increased anxiety-like behavior (Huang et al., 2025). VNUT is known to load ATP into astrocytic lysosomes and mediate its exocytotic release. Astrocyte-specific deletion of the glucocorticoid receptor gene (*Nr3c1*) via injection of GFAP-Cre-expressing adeno-associated virus (AAV-GFAP-Cre) into the mPFC of Nr3c1^loxP/loxP^ mice induces depressive-like behaviors by impairing lysosomal exocytosis-mediated ATP release, driven by the disruption of the phosphoinositide 3-kinase (PI3K)-Akt signaling pathway (Lu et al., 2022). Furthermore, mice lacking inositol 1,4,5-trisphosphate receptor type 2 (IP3R2^−/−^ mice), which is selectively found in astrocytes and mediates intracellular Ca^2+^ increases, show impaired calcium-dependent release of ATP from astrocytes and exhibit a depressive-like phenotype (Cao et al., 2013).

#### 2.2.2 Purinergic P2X7 receptors on astrocytes

The ATP-gated ion channel P2X7 receptor (P2X7R), a member of the ATP-sensitive P2XR superfamily, triggers the transmembrane flux of the small cations Na^+^, Ca^2+^, and K^+^. In the central nervous system (CNS), P2X7R is mainly expressed in microglia and, to a lesser extent, in neurons, astrocytes, and oligodendrocytes (Wei et al., 2025). Astrocyte-specific knockdown of P2X7Rs, achieved via intracerebroventricular delivery of AAV-GFAP-short hairpin RNA (shRNA), significantly exacerbates depression-like behaviors in the tail suspension test and forced swim test—hallmarks of depressive-like reaction to acute stressors (Cheng et al., 2025). Importantly, this behavioral phenotype is specific to astrocytic P2X7Rs.

#### 2.2.3 ATP-sensitive potassium channels on astrocytes

The K-ATP channels open when cellular ATP levels fall. The Kir6.1-containing ATP-sensitive potassium (Kir6.1/K-ATP) channels is prominently expressed in astrocytes and acts as metabolic stress sensor in the brain. The astrocyte-specific Kir6.1/K-ATP knockout (Kir6.1^loxp/loxp^; GFAP-Cre) mice exhibit aggravated depression-like behavior in chronic stress models, such as chronic unpredictable mild stress and chronic social defeat stress (Li et al., 2022). Kir6.1 deficiency under stress condition increases mitochondrial reactive oxygen species production and promotes NOD-, LRR-, and pyrin domain-containing protein 3 (NLRP3) inflammasome-dependent pyroptosis, an inflammatory form of programmed cell death (Li et al., 2022; Shi et al., 2015). Another K-ATP channel, the Kir6.2, is primarily located in neurons but is aberrantly expressed in reactive astrocytes during neuroinflammation (Griffith et al., 2016). The Kir6.2/K-ATP knockout mice exhibit aggravated depressive-like behaviors under chronic stress, accompanied by the loss of CA3 hippocampal neurons and the reduction of BDNF in the hippocampus. Notably, a deficiency in Kir6.2 results in antidepressant-like behaviors under non-stress conditions (Fan et al., 2016).

#### 2.2.4 Astrocytic sigma-1 receptors

The sigma-1 receptor (Sig-1R, SIGMAR1) is a molecular chaperone that is highly enriched at the endoplasmic reticulum (ER)—mitochondria contact sites in astrocytes. It regulates lipid metabolism, calcium signaling, the ER stress response, and BDNF release in astrocytes (Choi et al., 2022; Mysona et al., 2018). It is also involved in the maintenance of mitochondrial homeostasis. Thus, Sig-1R is a critical player in the cellular metabolism and function of astrocytes. Astrocyte-specific knockdown of Sig-1R in the ventral hippocampus—achieved by bilateral microinjection of AAV-GfaABC1D-shRNA—induces depressive-like behavior in mice (Li et al., 2025). Furthermore, siRNA-mediated knockdown of Sig-1R in primary astrocytes reduces intracellular ATP production but does not alter the mitochondrial membrane potential (Li et al., 2025).

#### 2.2.5 Insulin receptors in astrocytes

Insulin is an essential anabolic peptide hormone produced by the pancreas that regulates blood glucose levels. Insulin receptors (IRs) are widely distributed in the brain and play critical roles in regulating synaptic plasticity, cognitive function, and food intake (Blázquez et al., 2014). These receptors are found on both neurons and glial cells. Tamoxifen-inducible deletion of astrocytic IRs—achieved by crossing IR^flox/flox^ mice with GFAP-CreER^T2^ mice—causes increased depressive-like behaviors in the open field test, sucrose preference test, and forced swim test (Cai et al., 2018). Loss of astrocytic insulin signaling leads to impaired tyrosine phosphorylation of Munc18c, a critical regulator of SNARE-mediated membrane fusion. This impairment reduces exocytosis of ATP from astrocytes, resulting in decreased purinergic signaling on dopaminergic neurons. Because deleting astrocytic IRs in the nucleus accumbens (NAc) via AAV-GFAP-Cre infusion in IR^flox/flox^ mice partially reproduces these phenotypes, astrocytic insulin signaling may serve as a crucial bridge that utilizes ATP as a messenger to regulate dopamine (DA)-mediated neurotransmission in the brain.

### 2.3 Reduction of lactate production by lactate dehydrogenase A

Astrocyte metabolism centers on glucose, primarily using glycolysis to produce lactate. Astrocytes supply lactate to neurons as an energy substrate and modulate neuronal function. The astrocyte-specific deletion of the glycolytic enzyme lactate dehydrogenase A (LDHA), which converts pyruvate to lactate, can be achieved by injecting AAV-GFAP-Cre into the dorsomedial PFC of LDHA^flox/flox^ mice. These mice exhibit lower L-lactate levels, reduced excitability of dorsomedial PFC pyramidal neurons, and depression-like behaviors characterized by anhedonia-like behavior and behavioral despair (Yao et al., 2023).

### 2.4 Inability of calcium entry through astrocytic Orai1 channels

Orai1 is a crucial plasma membrane protein that forms the pore-forming subunit of store-operated Ca^2+^ channels, which are essential for bringing Ca^2+^ inside the cells. Store-operated calcium entry (SOCE) is a unique cellular process where the depletion of internal calcium stores in the ER triggers calcium influx from outside the cell. Indeed, Orai1 in astrocytes is activated by depleted internal calcium stores, which drives sustained calcium signals and gliotransmitter release, impacting synaptic function and behavior. Astrocyte-specific knockout of Orai1 channels using Orai1^flox/Y^; Aldh1l1-CreER^T2^ mice and tamoxifen abolishes SOCE in astrocytes, downregulates genes related to inflammation, metabolism, and the cell cycle, and induces metabolic reprogramming, including reduced levels of cellular metabolites and ATP/ADP (Novakovic et al., 2023). Astrocyte-specific loss of Orai1 also blunts Ca^2+^ signaling and inhibitory synaptic transmission and alters astrocyte function, which ameliorates lipopolysaccharide (LPS)-evoked depression-like behaviors (Novakovic et al., 2023).

### 2.5 Dysfunction of astrocytic Kir4.1 potassium channel

Kir4.1 channels are inward-rectifier potassium channels expressed exclusively in astrocytic processes. While Kir6.1 forms part of the metabolic stress-sensitive K-ATP channel, which regulates astrocytic pyroptosis and stress response, Kir4.1 serves as a primary channel for potassium homeostasis by mediating spatial potassium buffering, allowing astrocytes to take up excess extracellular potassium produced by neurons, thereby preventing neuronal hyperexcitability. In rat models of depression induced by the intraperitoneal injection of LPS, upregulated expression of Kir4.1 is observed in the lateral habenula (LHb), a brain region associated with aversion and negative emotions (Cui et al., 2018). These mice exhibit increased astrocyte membrane hyperpolarization, driven by increased potassium conductance, which triggers neuronal bursting. Supporting these observations, astrocyte-specific knockdown of Kir4.1—achieved by infusing AAV-shRNA or dominant-negative Kir4.1 into the LHb—reduces neuronal bursting and causes a pronounced reduction in depression-like phenotypes, including decreased immobility in the forced swim test and increased sucrose preference (Cui et al., 2018). Furthermore, these behavioral improvements can be reversed by overexpressing Kir4.1 via infusion of AAV-gfaABC1D-Kir4.1 into the LHb.

### 2.6 Gap junction dysfunction in astrocytes

Connexins (Cx30 and Cx43) are primary proteins that form gap junctions to facilitate the direct exchange of ions, metabolites, and second messengers, mediating intercellular communication between astrocytes. In astrocytes, these gap junctions allow for the release of gliotransmitters that modulate synaptic transmission (Duarte et al., 2024). Astrocyte-specific knockdown of connexins Cx30 and Cx43, using AAV-shRNA, reduces the frequency of spontaneous excitatory postsynaptic currents in pyramidal neurons of the mPFC and hippocampus, and elicits depression-like behavior (Huang et al., 2019). Furthermore, pharmacological blockade of gap junctions with carbenoxolone in the prelimbic region of the PFC reduces sucrose preference, an indicator of depression-like behavior (Sun et al., 2012). Similarly, injection of Cx43 mimetic peptides Gap27 or Gap26 into the PFC reduces sucrose preference (Sun et al., 2012).

## 3 Gain-of-function studies enhancing astrocyte functions

**Figure 3:**
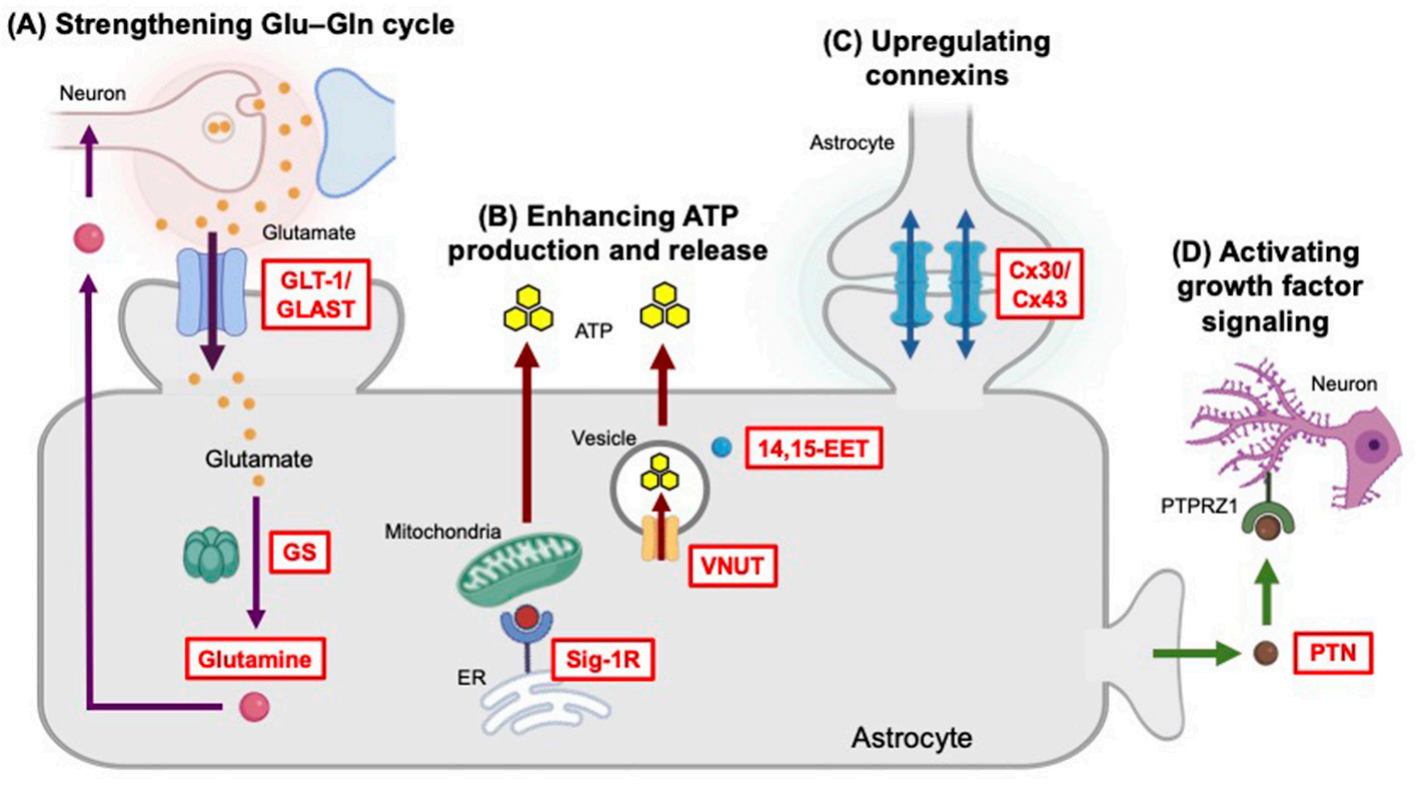
Methods to overcome depression-like phenotypes identified from GOF studies. **(A)** Enhancing the clearance of Glu from the synaptic cleft and strengthening the Glu-Gln cycle are effective strategies to reduce excitotoxicity and improve neuroplasticity. Upregulation of GLT-1 function can be achieved by inactivating ALKBH5, inhibiting OGT, or pharmacological manipulation, such as ceftriaxone and riluzole. Promotion of GS denitration and Gln supplementation also helps restore the reduced Glu/Gln levels, enhance neurotransmission, and alleviate stress-induced depression. **(B)** The production of ATP in astrocytes is enhanced by the activation of Sig-1R. The combined action of activated VNUT and high 14,15-EET levels boosts the release of ATP from astrocytes into the extracellular space, which act as an antidepressant mechanism by promoting neuroplasticity and neural repair through purinergic signaling. **(C)** Stabilization of connexins restores astrocyte network communication, allowing for the re-establishment of synaptic activity. **(D)** Astrocyte-derived growth factor PTN interacts with its receptor PTPRZ1 on excitatory neurons, subsequently activating the AKT signaling to regulate mood and behavior.

The GOF studies introduced in this chapter define how enhancing astrocytic functions—including Glu–Gln metabolism, purinergic signaling, metabolic signaling, and trophic factor pathways—ameliorates stress-induced behavioral deficits to overcome depression-like phenotypes in animal models (Figure 3 and Table 1).

### 3.1 Enhancement of the Glu–Gln cycle and Glu/Gln transporter function

Astrocytes play a pivotal role in maintaining extracellular Glu and Gln homeostasis via their regulation of Glu-to-Gln conversion and transport; they utilize Glu transporters (GLT-1/GLAST) for Glu uptake and sodium-coupled neutral amino acid transporters (SNAT3 and SNAT5) for Gln release.

#### 3.1.1 Upregulation of glutamate transporter GLT-1 and GLAST

The astrocytic glutamate transporter GLT-1 and GLAST is indispensable for clearing Glu from the synaptic cleft to prevent the brain from excitotoxic damage. Both the GLT-1 and GLAST immunoreactivity is found to be lower in postmortem tissue of patients with MDD (Miguel-Hidalgo et al., 2000). Lentiviral overexpression of GLT-1 and GLAST in the ventral hippocampus of high-fat diet (HFD)-induced depressed mice normalizes hyperactive glutamatergic transmission to the NAc and reduces depression-like behaviors (Tsai et al., 2022). Interestingly, chronic administration of the β-lactam antibiotic ceftriaxone in mice increases GLT-1 expression and produces antidepressant-like effects, manifested as reduced immobility time in the tail suspension test and forced swim test. The FDA-approved drug riluzole, which modulates Glu release and facilitates Glu clearance, is also known to increase GLT-1 mRNA expression (Banasr et al., 2010). In rats exposed to chronic unpredictable stress (CUS), riluzole reverses stress-induced anhedonia-like behavior in the sucrose preference test and helplessness in the active avoidance test. Riluzole also normalizes CUS-induced reductions in GFAP mRNA levels and mitigates HFD-induced depressive phenotypes by restoring the expression of GLT-1 and GLAST (Banasr et al., 2010).

Increased expression of GLT-1 in astrocytes is also reported in the mPFC of m6A RNA demethylase ALKBH5-deleted mice, achieved by using pLenti-GFAP-Cre virus in ALKBH5^loxP/loxP^ mice (Guo et al., 2024). N6-methyladenosine (m6A) is the most prevalent and abundant mammalian RNA modification, and ALKBH5 acts as an m6A eraser on target mRNA. Selective deletion of ALKBH5 in astrocytes increases m6A methylation of the GLT-1 mRNA in mPFC astrocytes, which helps clear excess Glu and protects against stress-induced synaptic dysfunction, neuronal atrophy, and depression-related behaviors.

In addition, GLT-1 is found to be O-GlcNAcylated by O-GlcNAc transferase (OGT), a key enzyme that not only catalyzes protein posttranslational modifications by O-GlcNAc but also acts as a stress sensor (Fan et al., 2023). Chronic stress-induced elevation of OGT in the mPFC leads to increased O-GlcNAcylation of GLT-1, which reduces Glu clearance from excitatory synapses. In contrast, selective deletion of astrocytic OGT in the mPFC of OGT^flox/Y^; CaMKIIa-CreER^T2^ mice, via bilateral injection of AAV-gfaABC1D-GFP-iCre, modulates glutamatergic synaptic transmission, preserves neuronal morphology, and ameliorates Ca^2+^ activity deficits caused by chronic stress, resulting in antidepressant-like phenotypes. Therefore, preventing the OGT-mediated downregulation of astrocytic GLT-1 contributes to antidepressant actions. In conclusion, evidence from these studies indicates that the upregulation of astrocytic Glu transporters contributes to antidepressant actions.

#### 3.1.2 Potentiation of glutamine synthetase

Glutamine synthetase (GS) is an essential enzyme that converts Glu to Gln. In the brain, GS is particularly observed in astrocytes and catalyzes the condensation of ammonia and Glu—both of which are neurotoxic—to synthesize Gln. GS immunoreactivity is significantly lower in postmortem tissue of MDD patients (Bernstein et al., 2015), and hypoactive GS has been shown to cause depressive behaviors. Indeed, the inhibition of GS activity via intraperitoneal injection of methionine sulfoximine reduces both Glu and Gln levels in the mPFC and induces depressive-like behaviors in mice (Son et al., 2018). Under chronic immobilization stress (CIS), where GS levels remain unchanged, the mice exhibit a depression-like phenotype alongside with reduced enzymatic activity of GS due to nitration of tyrosine (Tyr) residues (Kang et al., 2025). Tyr-nitration of GS is considered a natural inhibitory mechanism of GS activity, a process also observed in diseases such as epilepsy and hyperammonemia (Hakvoort et al., 2017; Qvartskhava et al., 2015). Pharmacological strategies that promote GS denitration have been proposed as novel antidepressant interventions. Dietary supplementation with Tyr significantly increases GS activity in the mPFC of CIS mice and produces antidepressant-like effects, as evidenced by improvements in the open field test, tail suspension test, and sucrose preference test. Furthermore, dipeptides such as tyrosyl–glutamine (YQ) can be supplemented in diets at higher concentrations than the single amino acid Tyr, achieving more robust denitration of GS and stronger antidepressant effects (Kang et al., 2025). GS activation by YQ is accompanied by the restoration of Glu and Gln levels and enhanced synaptic transmission in glutamatergic neurons. These results suggest that GS-mediated antidepressant effect can be enhanced by regulating Tyr-nitration in GS.

#### 3.1.3 Activation of amino acid transporter SNAT3 and SNAT5

The Gln synthesized in astrocytes by GS is reconveyed to presynaptic glutamatergic neurons by SNAT3 and SNAT5 in astrocytes and SNAT1 and SNAT2 in neurons. Although the function of SNAT3/5 in MDD pathogenesis is not fully understood, the simultaneous inactivation of SNAT3/5—via injection of Cre recombinase and small guide RNA-encoding viruses into CRISPR/CAS9-EGFP mice—induces depressive-like behaviors with reduced Glu/Gln levels in mice (Kang et al., 2026). Similarly, CIS-induced depression in mice also reduces SNAT3/5 expression in the mPFC (Baek et al., 2019). Of note, the individual deletion of SNAT3 and SNAT5 have little effect on emotional behavior. Interestingly, Gln supplementation in diet shows antidepressant effects against CIS, along with the recovery of SNAT3/5 expression and increased level of Glu/Gln in the mPFC (Baek et al., 2019; Son et al., 2018). Thus, the efflux of Gln from astrocytes via SNAT3/5 is necessary for maintaining Glu-Gln cycle in the mPFC.

### 3.2 Potentiation of purinergic and metabolic signaling

Astrocytes release ATP, which is rapidly degraded to adenosine, forming a purinergic extracellular signaling system. This system is crucial for brain homeostasis. Astrocytic mitochondria are key metabolic signaling hubs, as they control ATP supply, neurotransmitter balance, and redox state. Furthermore, astrocytic lipid metabolism is important for crosstalk with neurons.

#### 3.2.1 ATP and adenosine upregulations

Insufficient ATP release and impaired ATP signaling is shown to associate with depression-like behavior. Indeed, a dysregulation of genes involved in the biosynthesis of ATP has been observed in the mPFC of the MDD patients (Klempan et al., 2009). In agreement with the role of astrocytic ATP in mood regulation, stimulating ATP release from astrocytes or infusing ATP in the brain has been shown to have antidepressant effects. The selective serotonin reuptake inhibitor fluoxetine enhances astrocytic ATP exocytosis in a vesicular nucleotide transporter (VNUT)-dependent manner, thereby mediating antidepressant effects. Indeed, astrocyte-specific overexpression of VNUT, using the double-transgenic mice derived from astrocyte-specific tetracycline trans-activator (tTA) lines (Mlc1-tTA lines) and VNUT tetO knockin lines, significantly potentiates fluoxetine-induced ATP release. Conversely, VNUT knockout mice exhibit a significantly weaker response to fluoxetine (Kinoshita et al., 2018). ATP and its metabolite adenosine, act on both astrocytic P2Y11 receptors and adenosine A_2_B receptors, leading to activation of the cAMP/PKA pathway, which results in the upregulation of BDNF mRNA expression, a proposed marker for MDD treatment response (Cavaleri et al., 2023). The administration of ATP can alleviate depressive-like behavior in mice. Notably, the intracerebroventricular infusion of both ATP and ATP-γ-S, a non-hydrolyzable ATP analog, induces antidepressant-like effects (Cao et al., 2013; Wang et al., 2025).

#### 3.2.2 Activation of Sig-1R

The Sig-1R is a molecular chaperone that is highly enriched at the ER-mitochondria contact sites in astrocytes and regulates calcium signaling, lipid metabolism and the ER stress response. It is also involved in the maintenance of mitochondrial homeostasis. Sig-1R has been suggested as a contributor to MDD. Activation of astrocytic Sig-1R is sufficient to ameliorate inflammation-induced depression (Guo et al., 2021; Yang et al., 2019). In an LPS-induced depression model, AAV-GFAP-Sig-1R-induced astrocyte-specific overexpression of Sig-1R significantly reduces the expression of the astrocyte activation marker GFAP, increases BDNF, and attenuates the LPS-induced increase in mRNA levels of inflammatory mediators such as TNF-α, IL-1β, and iNOS (Guo et al., 2021). The rapid antidepressant action of the novel agent YL-0919 is also mediated by astrocytic Sig-1R (Li et al., 2025). Activation of Sig-1R by YL-0919 increases intracellular ATP production and mitochondrial membrane potential in astrocytes, whereas these effects are abolished by Sig-1R knockdown. Moreover, YL-0919 enhances the expression of pro-BDNF and mature BDNF in a Sig-1R-dependent manner, and this upregulation can be blocked by Sig-1R knockdown.

The antidepressive action of Sig-1R does not seem to be restricted to astrocytes. Recent findings suggest that ketamine, a glutamate NMDA receptor blocker known for its rapid and sustained antidepressant effects, activates the mitochondrial inner membrane protein TAMM41. This activation promotes a TAMM41-cardiolipin-exosome axis that facilitates the transfer of astrocyte-derived Sig-1R to neurons (Guo et al., 2025). Astrocyte-specific conditional deletion of TAMM41 by injecting AAV-GFAP-Cre into TAMM41^flox/flox^ mice, induces depression-like behavior and abolishes the long-lasting antidepressant effects of ketamine (Guo et al., 2025). However, the exogenous administration of exosomes encapsulating Sig-1R mRNA produces rapid and sustained antidepressant-like effects and improves neuronal morphology, including increased neurite length and branching. These results suggested that the antidepressive action can be achieved by targeting organelle proteins in astrocytes that regulate cellular metabolisms.

#### 3.2.3 Elevation of fatty acid binding protein 7 expression

Fatty acid binding protein 7 (FABP7) is a lipid metabolism-related protein highly expressed in hippocampal astrocytes. Bilateral injection of AAV-mediated overexpression of FABP7 in the hippocampus attenuates depression-like behavior in mice, as shown by improvements in the forced swim test, tail suspension test, and sucrose preference test. These effects are mediated through the modulation of lipid metabolism, neuroinflammation, and blood brain barrier (BBB) stability (Geng et al., 2025). FABP7 GOF upregulates the downstream caveolar structural protein caveolin-1 and suppresses neuroinflammatory responses. It also increases hippocampal BDNF levels, restores dendritic spine density, and normalizes the expression of postsynaptic proteins such as post synaptic density protein 95 (PSD95) and glutamate AMPA receptor GluA1 subunit. In addition, FABP7 overexpression reverses reductions in BBB-associated proteins, including the tight junction components claudin-5 and occludin, and the water channel AQP4, induced by chronic unpredictable mild stress. Collectively, these findings indicate that FABP7 exerts antidepressive action by improving astrocytic homeostasis and neural plasticity via lipid metabolic pathways.

#### 3.2.4 Enhancement of astrocytic epoxyeicosatrienoic acid signaling

Astrocytes exhibit increased fatty acid metabolism compared with other neural cell types (Cahoy et al., 2008). Epoxyeicosatrienoic acids (EETs), which are bioactive signaling molecules in astrocytes produced from arachidonic acid by cytochrome P450 epoxygenase enzymes in response to Glu, are known to play a key role in neurovascular coupling. Reduced expression of genes involved in EET signaling is found in both MDD patients and in the mPFC of mice subjected to chronic social defeat stress (Xiong et al., 2019). An infusion of soluble epoxide hydrolase (sEH) inhibitors, which inhibit the degradation of EETs, into the mPFC of mice produces antidepressant-like phenotypes, specifically by reducing the duration of immobility in the forced swim test without affecting locomotor activity. Similarly, the direct infusion of EETs, in the form of 14,15-EET, into the mPFC produces an antidepressant-like phenotype. The specific deletion of *Ephx2* (which encodes sEH) in astrocytes produces a rapid antidepressant-like effect, coupled with elevated ATP release. These results suggest that EET signaling in the mPFC is essential for the expression of depressive-like behaviors by regulating ATP release from astrocytes.

### 3.3 Upregulation of connexins

Connexins (Cx30 and Cx43), which form gap junctions and release gliotransmitters, contribute to mood regulation. In the mPFC and hippocampus of mice exposed to chronic social defeat stress, the expression of astrocytic connexins is significantly reduced, and lower neuronal activity is observed (Huang et al., 2019; Wang et al., 2025). In contrast, the overexpression of astrocytic connexins in the mPFC and hippocampus—achieved by the injection of AAV into GFAP-Cre/Thy1-EGFP mice—increases neuronal activity and suppresses depression-like behavior (Huang et al., 2019).

Astrocytic mitochondria also express Cx43 in both monomeric and polymeric forms and contribute to the maintenance of mitochondria homeostasis. Overexpression of mitochondrial Cx43 in astrocytes—achieved by injecting a vector encoding the Cx43 gene fused to a mitochondrial targeting sequence—rescues neuronal excitability and mitigates depressive-like behaviors. This effect is possibly through the interaction with isocitrate dehydrogenase 3a, which restores oxidative phosphorylation and ATP production, while also impacting glucose uptake and glycolysis (Ye et al., 2025). Together, these findings indicate that astrocytic connexins support neuronal excitability and resilience to stress.

### 3.4 Enhancement of astrocyte-derived growth factor signaling

Astrocytes are found to exert antidepressant actions through trophic signaling mechanisms that modulate synaptic plasticity and neuronal resilience. Recent work has identified pleiotrophin (PTN), an astrocyte-derived growth factor also known as heparin-binding growth-associated molecule, as a critical regulator for stress susceptibility in the PFC (Chi et al., 2025). Single-nucleus transcriptomic analyses reveal a reduced number of astrocyte and attenuated signaling between PTN and its receptor, protein tyrosine phosphatase receptor type Z1 (PTPRZ1), in MDD patients. Astrocyte-specific PTN knockdown—achieved by bilateral injection of AAV-DIO-PTN-shRNA into the dorsomedial PFC of tamoxifen-administered Aldh1l1-CreER^T2^ mice—induces depressive-like behaviors, whereas PTN overexpression in the dorsomedial PFC of Aldh1l1-CreER^T2^ mice by the injection of AAV or exogenous PTN supplementation reverses these deficits (Chi et al., 2025). The antidepressant effects of PTN require its receptor PTPRZ1 on excitatory neurons, whereby PTN–PTPRZ1 signaling activates the AKT pathway to promote neuronal stability and circuit-level plasticity.

## 4 Linking astrocyte dysfunction and depressive behavior: disruption of neural circuits

As found in Chapters 2 and 3, astrocytic dysfunction is strongly linked to the pathophysiology of depression. This chapter discusses how such cellular-level abnormalities are integrated into disruptions of major neural circuits in the CNS, particularly those governing reward processing and stress responses, through underlying molecular mechanisms.

### 4.1 Impaired mesolimbic reward processing and anhedonia

Anhedonia, a core symptom of depression, is closely associated with dysfunction of the mesolimbic reward circuit, characterized by dopaminergic projections from the ventral tegmental area (VTA) to the NAc (Cai et al., 2018; Huang et al., 2025; Yao et al., 2023). Astrocytic dysfunction influences reward-related behaviors primarily by modulating DA signaling within the NAc. Astrocyte-specific deletion of the VNUT, which is involved in the exocytotic release of ATP, significantly reduces basal extracellular DA levels in the NAc. This reduction leads to decreased reward motivation, as assessed by progressive ratio breakpoints, and the emergence of depression-like behaviors (Cai et al., 2018; Huang et al., 2025). These findings suggest that astrocyte-derived ATP positively regulates DA release in the NAc via purinergic P2Y receptors. Astrocytic IR also serves as critical regulators of reward processing, directly linking metabolic signaling to the development of anhedonia. Reduction of astrocytic IRs diminishes exocytosis of ATP via a specific SNARE complex containing Munc18c, resulting in decreased purinergic signaling on dopaminergic neurons in the NAc and VTA (Cai et al., 2018).

Furthermore, inhibition of the astrocytic GLT-1 in the PFC, particularly within the IL cortex, induces anhedonia-like behavior, characterized by elevated intracranial self-stimulation thresholds in response to medial forebrain bundle stimulation, occurring independently of other anxiety-related phenotypes (Fullana et al., 2019; John et al., 2012). O-GlcNAcylation—the OGT-mediated posttranslational modifications of GLT-1 in astrocytes—reduces GTL-1 functions, thereby diminishing the transporter's ability to clear Glu from the synaptic cleft. This leads to aberrant glutamatergic signaling and neuronal atrophy in the mPFC (Fan et al., 2023). Because glucose is used to produce UDP-GlcNAc, which acts as a donor molecule for O-GlcNAcylation (Fan et al., 2021), O-GlcNAcylation is a nutrient-sensitive modification, serving as a critical molecular bridge between metabolism and behavioral states such as anhedonia, depression, and reward processing. Consequently, disruption of Glu homeostasis in the PFC is thought to suppress downstream reward signaling to the NAc.

### 4.2 Disruption of prefrontal-amygdala circuits and abnormal stress responses

The astrocyte-derived trophic factor PTN interacts with the receptor protein tyrosine phosphatase PTPRZ1 in neurons and activates AKT signaling pathways (Chi et al., 2025). PTN deficiency in the PFC results in reduced synaptic density, reflected by decreased PSD95 expression, and diminished neuronal firing frequency, accompanied by depression-like behaviors. These findings indicate that astrocytes support proper PFC function by maintaining excitatory synaptic transmission through PTN-dependent mechanisms.

The PFC plays a central role in the top-down regulation of stress responses, and the dysfunction within limbic structures such as the hippocampus and amygdala is strongly associated with the development of depression and comorbid anxiety (Codeluppi et al., 2023; John et al., 2015). Research indicates the blockade of the GLT-1 in the PFC not only induces anhedonia-like behavior but also triggers widespread activation of limbic regions, including the central and basolateral amygdala, as evidenced by increased c-fos expression (John et al., 2012). Thus, local impairment of astrocytic Glu uptake likely contribute to hyperexcitability within these limbic circuits.

## 5 Remaining questions

The LOF/GOF studies in astrocytes strongly support the role of astrocyte dysfunction in depression (Table 1). Furthermore, several astrocytic functions, such as maintenance of the Glu–Gln cycle, ATP exocytosis, and the exchange of ions and second messengers through gap junctions, are highlighted as critical regulator of mood (Figure 1). However, it remains unclear how region-specific astrocytic alterations are integrated across neural circuits and whether recently identified intercellular and inflammatory mechanisms represent generalizable principles of depression.

### 5.1 Circuit-level integration of region-specific astrocytic pathology

It remains poorly understood how astrocytic dysfunction across distinct brain regions, including the PFC, hippocampus, NAc, and VTA, cooperates or antagonizes to shape the complex of depressive symptoms, such as anxiety and anhedonia. For example, the effects of astrocyte activation on dopaminergic neuron activity in the VTA must be integrated into a broader framework of large-scale networks that mediate emotional regulation. In addition, astrocyte-mediated regulation of neural activity in the LHb via Kir4.1 has emerged as a critical mechanism in depression (Cui et al., 2018). The LHb serves as a key hub that connects the forebrain to midbrain monoaminergic systems, integrating aversive information and translating it into negative emotions (Zhang et al., 2022). Further research is needed to determine if distinct subpopulation of LHb neurons project to different areas and are differentially regulated by Kir4.1.

### 5.2 Generality of epitranscriptomic and intercellular transfer mechanisms

The antidepressant effects of ketamine rely on the exosome-mediated transfer of astrocyte-derived Sig-1R to neurons (Guo et al., 2025), but it remains unclear whether similar intercellular transport mechanisms apply to other astrocyte-derived factors such as FABP7 or PTN, and to what extent defects in these pathways contribute to the pathogenesis of depression. In addition, the ALKBH5/m6A pathway in astrocytes has been identified as a crucial cell-autonomous regulator of depressive-like behavior through the m6A modification of GLT-1 (Guo et al., 2024). These findings suggest that astrocytic epigenetic changes may bridge the gap between the genetic and environmental factors in depression. Given that the m6A methyltransferases (METTL3, MTTL14, and WTAP) and the m6A demethylases (ALKBH5 and FTO) are altered in the patients with MDD (Ngo et al., 2025), further studies are needed to investigate their region-specific effects and targets.

### 5.3 Regulation of pyroptosis

The ablation of astrocytic Kir6.1-induced NLRP3-dependent pyroptosis has been shown to worsen depression-like behavior (Li et al., 2022), however, the dynamic molecular switches by which astrocytes modulate cell survival or death through changes in Kir channel activity under stress or inflammatory conditions are not fully characterized. Likewise, the downstream inflammatory cascade triggered by Orai1-mediated Ca^2+^ signaling requires further dissection.

## 6 Therapeutic implications and future directions

Astrocytes are now being regarded as active regulators of circuit function and promising therapeutic targets (Zhang et al., 2023). As summarized in Chapters 2 and 3, LOF/GOF studies have demonstrated that manipulating astrocytic function can bidirectionally modulate depression-like behavior (Table 1). However, most of these data are derived from rodent models, and the extent to which they can be translated into human MDD remains uncertain. In this chapter, we classify astrocyte-related targets according to their level of preclinical and translational support and outline future research directions.

### 6.1 Preclinical proof-of-concept targets

Several astrocyte-related pathways have now demonstrated robust preclinical proof-of-concept. These include (i) the Glu–Gln cycle (Baek et al., 2024; Blacker et al., 2019; Fontana, 2015), (ii) glycolytic/mitochondrial ATP production and lactate shuttling (Carrard et al., 2018; Chamaa et al., 2024; Jouroukhin et al., 2018; Yin et al., 2021), (iii) purinergic signaling and VNUT-dependent ATP release (Kinoshita et al., 2018; Koizumi, 2022), (iv) astrocyte-derived neurotrophic factors (Chi et al., 2025; Rial et al., 2016), and (v) lipid and BBB regulators, such as FABP7 (Bai et al., 2015; Geng et al., 2025). At the same time, issues such as brain-region specificity, chronic safety, and pharmacokinetics, including BBB permeability and cell-type selectivity, remain major obstacles to clinical translation.

### 6.2 Targets with an emerging translational bridge

A subset of astrocyte-related targets already has partial support from human studies or clinical pharmacology and thus lies closer to translational application. First, GLT-1 which is indirectly targeted by riluzole, a therapeutic agent for amyotrophic lateral sclerosis, has been reported to exert antidepressant effects in clinical trials in humans (Sanacora et al., 2007; Zarate et al., 2004). Although these compounds are not astrocyte-selective and have broad pharmacological profiles, their ability to enhance Glu uptake suggests that boosting astrocytic Glu clearance may have therapeutic value in humans.

The Sig-1R agonists such as YL-0919 represent another promising class of compounds. Preclinical studies indicate that astrocytic Sig-1R at ER–mitochondria contact sites is required for the rapid antidepressant effects of YL-0919 (Li et al., 2025; Wang et al., 2024), through enhancement of ATP production, mitochondrial membrane potential, and BDNF expression. Corroborating these reports, clinical data also suggest astrocytic Sig-1R is a partially validated target for depression (Malar et al., 2023; Safrany and Brimson, 2016).

Finally, several astrocyte-related biomarkers are emerging from human imaging and peripheral fluid studies. MRS studies provide non-invasive readouts of astrocyte-enriched metabolites, such as myo-inositol, Glx (Glu + Gln), and lactate, which are often altered in MDD (Block et al., 2009; Chen et al., 2009; Coupland et al., 2005; Moriguchi et al., 2019). PET tracers for monoamine oxidase-B, a marker of reactive astrocytes, have been validated in other CNS disorders, could in principle be applied to depression to visualize astrocyte activation (Cavaliere et al., 2020; Gill et al., 2022; Harada et al., 2022). In parallel, peripheral markers, including serum GFAP and S100B, as well as astrocyte-derived extracellular vesicles containing GFAP, AQP4, and inflammatory mediators, are being investigated as state or trait markers linked to astrocytic pathology (Schroeter et al., 2008; Singhal et al., 2025; Steinacker et al., 2021; Xu et al., 2023). Other astrocyte-associated molecules, such as NLRP3, Orai1, Kir4.1, Kir6.1, AQP4, FABP7, ALKBH5, and connexins have compelling mechanistic data in rodents but limited evidence in humans. These examples illustrate that a translational bridge from rodent models to human MDD is beginning to form, and systematically integrating astrocyte-focused pharmacology with imaging and blood-based biomarkers may enable stratification of patients according to glial phenotypes and facilitate mechanism-based clinical trials.

### 6.3 Future research directions

Moving astrocytes from “background contributors” to “bona fide therapeutic targets” in depression still require progress on several fronts.

- Refinement of human astrocyte pathology: Large-scale postmortem and single-cell/spatial transcriptomic studies are needed to delineate cell-type- and region-specific astrocyte alterations across cortical and subcortical circuits in MDD, and to relate this information to the genetic risk and clinical subtypes (e.g., treatment-resistant, psychotic, or high-suicide-risk depression).
- Development and validation of astrocyte-informed biomarkers: Longitudinal studies combining MRS, astrocyte-targeted PET, serum/CSF GFAP/S100B, and astrocyte-derived extracellular vesicles should clarify how glial states map onto symptom dimensions and treatment response. Such biomarkers could be used both for patient stratification and as pharmacodynamic readouts in clinical trials.
- Design astrocyte-directed pharmacological interventions: Based on preclinical LOF/GOF works, candidate strategies include GLT-1 enhancers, GS and LDHA modulators, agents that boost VNUT-dependent ATP release and astrocytic BDNF production, and Sig-1R agonists. Future compounds should be optimized for cell-type and brain-region selectivity and evaluated in combination with existing monoaminergic antidepressants.
- Circuit-level integration and behavioral relevance: Astrocyte-targeting interventions must be understood in terms of their impact on defined circuits as well as specific behavioral domains, including anhedonia, anxiety, and cognitive dysfunction. Cross-species approaches that align rodent circuit manipulations with human imaging and behavioral phenotyping are crucial.

In conclusion, astrocytes are emerging as central regulators of mood-related circuits and as attractive, though still largely experimental, targets for novel antidepressant therapies. Bridging detailed mechanistic insights from LOF/GOF studies with human biomarker and clinical intervention data will be key to realizing the therapeutic potential of astrocyte-focused strategies in depression.
